# Supplementary material for: A Sensitive Impedimetric Aptasensor Based on Carbon Nanodots Modified Electrode for Detection of 17ß-Estradiol
Source: Nanomaterials (Basel). 2020 Jul 10;10(7):1346. doi: 10.3390/nano10071346 (PMC7407411; doi:10.3390/nano10071346)
Supplement: Supplementary file 1 [file nanomaterials-10-01346-s001.pdf]

Supplementary Materials

A Sensitive Impedimetric Aptasensor Based on Carbon Nanodots Modified Electrode for Detection of 17ß-Estradiol

Mohd Hazani Mat Zaid [1], **Jaafar Abdullah** [3], **Normazida Rozi** [2], **Aliff Aiman Mohamad Rozlan** [2] and **Sharina Abu Hanifah** [1,2,*]

[1] Department of Chemical Sciences, Faculty of Science and Technology, Universiti Kebangsaan Malaysia, 43600 Bangi, Selangor, Malaysia

[2] Polymer Research Centre, Faculty of Science and Technology, Universiti Kebangsaan Malaysia, 43600 Bangi, Selangor, Malaysia

[3] Department of Chemistry, Faculty of Science, Universiti Putra Malaysia, 43400 UPM Serdang, Selangor Darul Ehsan, Malaysia

* Correspondence: sharina @ukm.edu.my

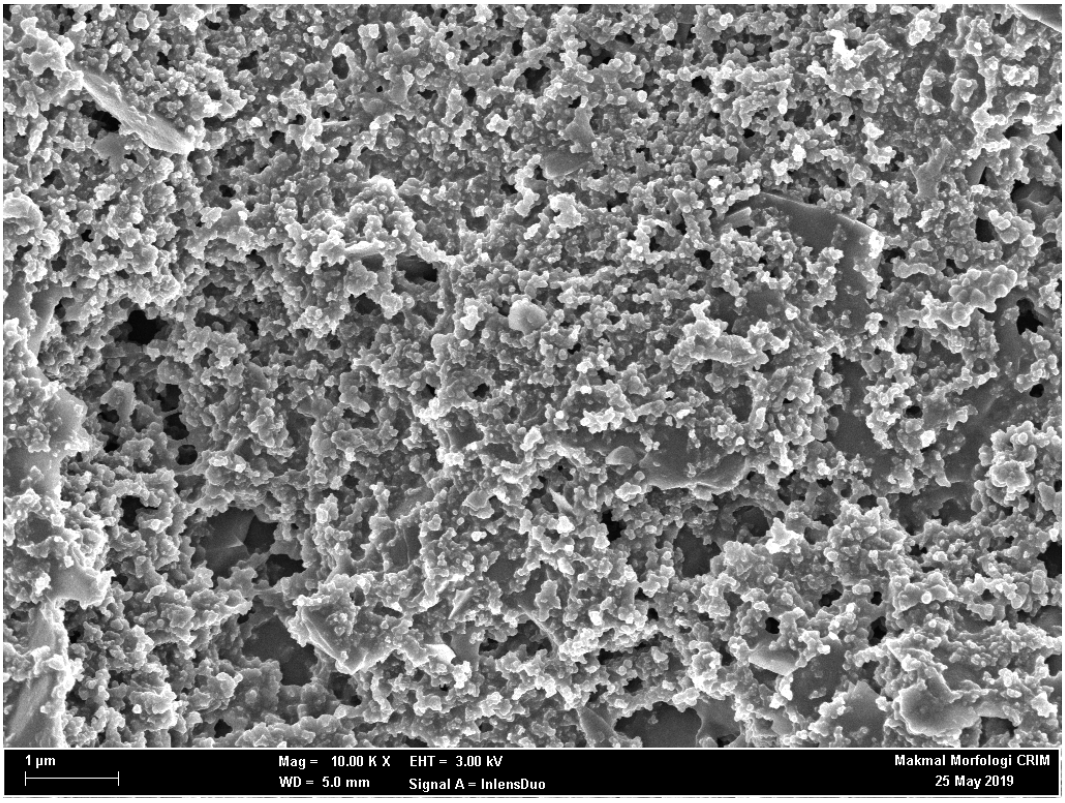

Figure S1. FESEM image for bare SPCE.

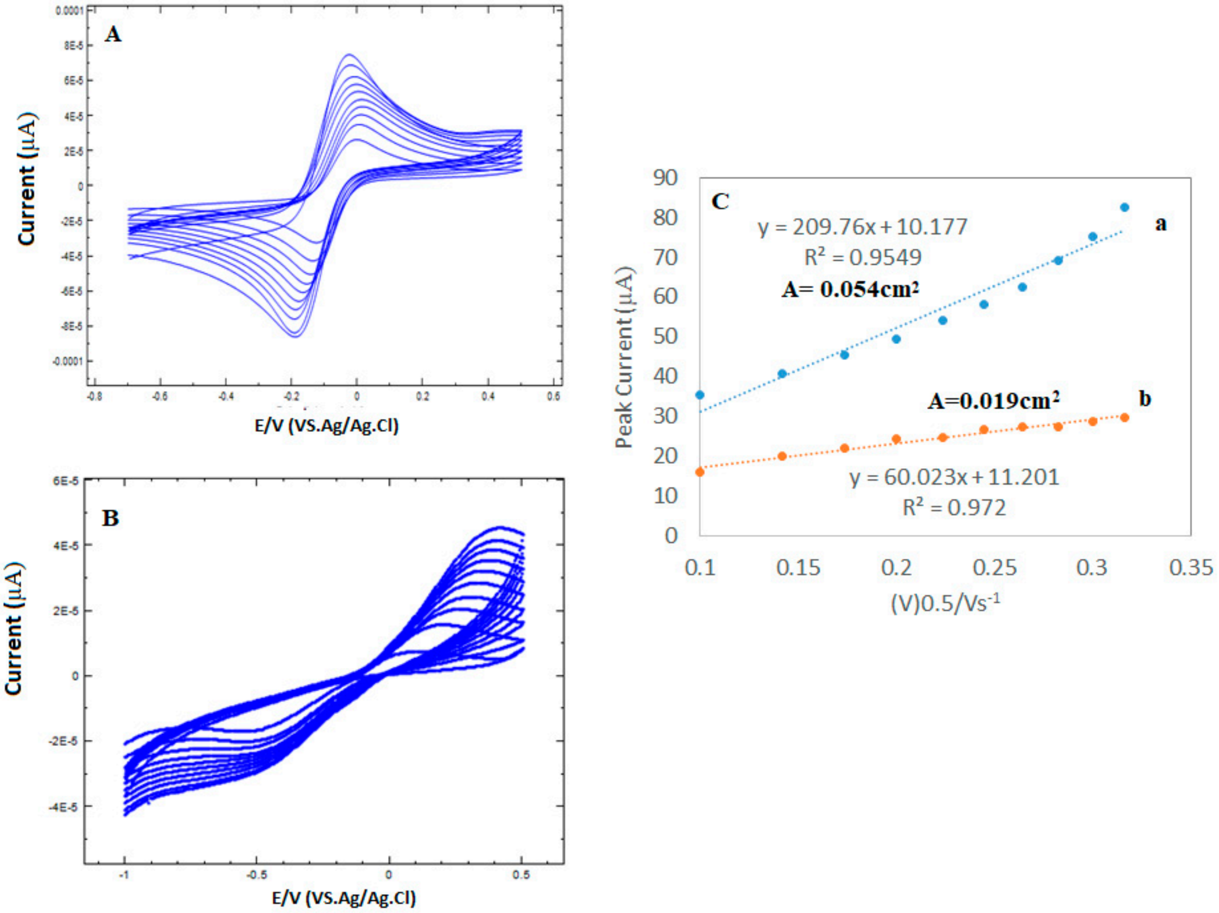

Figure S2. Cylic voltammogram of A) CDs/SPCE and B) Bare SPCE at different scan rate (10-100mVs^{-1}). Electroactive surface area of the electrode by CVs: (a) CDs/SPCE, and (b) Bare SPCE in 5.0 mM Fe $(CN)_6^{4-}$/ $K_4[Fe(CN)_6]^{3-}$ at different scan rates.
